# Orchiectomy Decreases Locomotor Activity and Delays the Expression of the Clock Protein PER1 in the Suprachiasmatic Nucleus in Rabbits

**DOI:** 10.3390/ani14243570

**Published:** 2024-12-11

**Authors:** Ángel Roberto Guzmán-Acevedo, Mario Daniel Caba-Flores, Rubi Viveros-Contreras, José Enrique Meza-Alvarado

**Affiliations:** 1 Programa de Doctorado en Ciencias Biomédicas, Universidad Veracruzana, Xalapa 91090, Veracruz, Mexico; racevedo0911@gmail.com; 2 Departamento de Bioquímica y Medicina Molecular, Facultad de Medicina, Universidad Autónoma de Nuevo León, San Nicolás de los Garza 64460, Nuevo León, Mexico; md_caba@hotmail.com; 3 Centro de Investigaciones Biomédicas, Universidad Veracruzana, Xalapa 91090, Veracruz, Mexico; ruviveros@uv.mx

**Keywords:** clock gene, hypothalamus, behavior, Cosinor

## Abstract

The suprachiasmatic nucleus (SCN) is the master regulator of the circadian system, modulating the daily timing of physiological and behavioral processes in mammals. While SCN synchronization is primarily driven by environmental light signals, sex hormones, particularly androgens, have a crucial role in regulating behavioral and reproductive processes to align with daily or seasonal cycles. SCN cell populations express receptors for sex steroid hormones, contributing to circadian synchronization mechanisms. Specifically, the activation of androgen receptors in the SCN has been shown to modulate clock gene expression and influence circadian rhythms. Rabbits, widely used in experimental research, exhibit unique behavioral patterns, including plasticity in circadian typology and seasonal variations in testosterone secretion. In this study, we explored, in male rabbits, the effect of castration on the daily pattern of locomotor activity and the expression of the clock protein PERIOD 1 (PER1) in the SCN. Our results show that castration significantly reduces daily locomotor activity and PER1 expression in the SCN. Moreover, a 4 h delay in the acrophase of PER1 expression was observed. We conclude that androgens have an important role in SCN synchronization mechanisms, contributing to the organization of physiological and behavioral events in this species.

## Simple Summary

The rabbit (*Oryctolagus cuniculus*), unlike primarily nocturnal rodents, exhibits flexible daily rhythms in its physiology and behavior, allowing it to adapt to nocturnal, diurnal or crepuscular conditions. These daily rhythms are regulated by a biological clock, a group of cells at the base of the brain. Ambient light is the main synchronizing signal of this clock, and through different pathways, time-related cues are communicated to other organs. There is a group of clock genes expressed at molecular level in every cell that contribute to modulating the daily rhythms throughout the body, and the endocrine system links the biological clock with the rest of the body. This study demonstrates how the absence of testicular hormones affects the expression of a clock protein and alters the daily pattern of locomotor activity, suggesting a clear endocrine effect on the biological clock and behavior. Our findings may relate to the seasonal reproductive behavior observed in this species.

## 1. Introduction

Circadian rhythms are behavioral and physiological cycles that repeat at intervals of approximately 24 h. In mammals, the suprachiasmatic nucleus (SCN) is the master clock coordinating these rhythms. The SCN is primarily synchronized by environmental light cues, although other signals such as feeding and locomotor activity are also important. The molecular control of circadian rhythms involves a positive and negative feedback loop of transcription and translation, regulated by a group of genes named clock genes and their proteins. The CLOCK and BMAL1 proteins form a dimer that initiates the transcription of *Per1*, *Per2*, *Per3*, *Cry1*, *Cry2*, *Rev-erb*, and *Ror* genes. The PER and CRY proteins form dimers that bind to CLOCK:BMAL dimers, inhibiting their own transcription [1,2]. During the oscillation of these gene expressions, other genes named clock-controlled genes are induced, many of which are involved in key physiological processes [3]. The SCN communicates with central and peripheral tissues through two pathways: direct afferent and efferent projections [4,5] and a humoral pathway through the neuroendocrine system [6]. Androgen receptor (AR) expression in the SCN has been demonstrated in several species, including rats [7], mice [8], and humans [9]. This suggests that ARs modulate circadian rhythms related to gonadal hormone secretion, potentially providing feedback to regulate their own physiology [10,11]. Early studies in animals such as the hamster (*Mesocricetus auratus*; [12]), diurnal rodent (*Octodon degus*; [13]), mouse (*Mus musculus*; [10]), and rat (*Rattus norvergicus*; [14]) reported chronobiological and behavioral changes following surgical removal of the testicles, which were attributed to reduced systemic androgen release.

Studies in rodents suggest that androgens alter the SCN response to light, as well as the expression of clock genes, and influence behavioral responses to different phases of the photoperiod [15]. Reports in mice have demonstrated the presence of ARs in the SCN [10], specifically in a retinorecipient region of the nucleus, in gastrin-releasing peptide cells. These cells receive two types of input: a direct input through the retinohypothalamic tract, and an indirect input via a humoral route through the secretion of androgens [10]. Hence, androgens are important in the regulation of the SCN circuit [16]. The New Zealand White rabbit is a widely used animal in laboratory studies and biomedical research [17]. It has been reported that these rabbits exhibit well-established daily rhythms in locomotor activity [18] with either nocturnal or diurnal patterns [19,20,21], Additionally, the expression of the clock protein PER1 in the SCN has been well documented [21,22]. However, in male rabbits, testosterone is released into the bloodstream in ultradian pulses, with five to six peaks per day [23]. Also, plasma concentrations are higher in summer, influencing the secretion of luteinizing hormone-releasing hormone. Therefore, based on these observations, androgens could influence the daily rhythm of locomotor activity and the expression of the clock protein PER1 in the SCN of the rabbit.

The objective of this study was to investigate the effect of castration on the daily pattern of locomotor activity and the expression of the clock protein PER1, one of the most important elements of the circadian clock in the SCN of mammals [24].

## 2. Materials and Methods

### 2.1. Animals and Housing Conditions

Forty-eight male New Zealand White rabbits from our colony in Xalapa, Mexico (located at the coordinates 19°30′59.3″ N 96°52′26.9″ W) were used for the experiments. The rabbits were maintained in a controlled environment with a 12 h light/dark cycle (lights on at 7:00 h, designated as zeitgeber time 0, ZT0) at 300 lux and a controlled temperature of 23 °C ± 2 °C. Filtered water and food pellets (Purina®, Cuautitlán, México) were available ad libitum. The food contained 12% moisture, 14.50% protein, 2% fat, 1% calcium, 18% fiber, 10% minimum ash, 43.50% FNE, 0.45% phosphorus, and added vitamins and was free of antibiotics. At eighteen weeks of age, the rabbits were placed in Omnialva^®^ SICARD (Ciudad de Mexico, Mexico) register boxes and underwent an adaptation period of seven days. Each rabbit was housed individually for monitoring of their locomotor activity, with each cage containing a compartment (0.60 cm wide × 0.50 cm long × 0.40 cm high). The activity of the rabbits was monitored by an infrared radiation-sensitive detector (λ5–14 μm) located on the ceiling of the box. This device contains a pyroelectric sensor coupled to a Fresnel lens (Parallax^®^ cat. 28027; Rocklin, CA, USA) that detects animal movements through the infrared radiation emitted by their bodies. When subjects move, a signal is generated by the sensor, transduced, stored in 15 s bins, and converted to 4 h bins for analysis.

**Figure 1:**
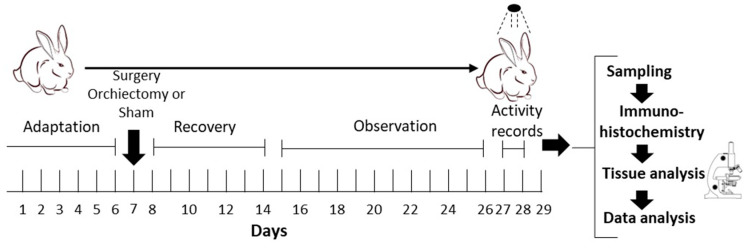
Experimental design.

For the gonadectomized group (GDX *n* = 24), rabbits were anesthetized and underwent gonadectomy. Following surgery and a six-day recovery period, locomotor activity was recorded. The data considered for the analysis were from days 27 and 28. After this period, the rabbits were perfused at 4 h intervals over a 24 h cycle (*n* = 4 per interval), starting at ZT03 (10:00 h). In the control group (Sham *n* = 24), rabbits were housed and manipulated like the GDX, including surgery without the removal of the testicles. The control animals were also given a 6-day recovery period before resuming locomotor activity monitoring (Figure 1). The surgeries were performed by a specialized veterinarian. All experimental procedures were carried out in accordance with the procedures of the National Guide for the Production, Care, and Use of Laboratory Animals (Norma Oficial Mexicana NOM-062-ZOO-1999) [25], which complies with the International Guidelines laid down by the Society for Neuroscience, and approved by the technical committee of the Biomedical Research Center, No. 005/2012.

### 2.2. Gonadectomy

Anesthesia was administered using an intramuscular injection of ketamine hydrochloride (60 mg/kg) combined with xylazine (8 mg/kg). After preparation of the area, and under aseptic conditions, the testicles were externalized via a scrotal midline surgery and removed after clamping the testicular artery. The incision was sutured with No. 0 chromic catgut, and a topical antibiotic (nitrofurazone 0.2 g) and the administration of flunixin meglumine (1 mg/kg I.M.) were used as a preventive treatment.

### 2.3. Immunohistochemistry

At day 27, an overdose of sodium pentobarbital (60 mg/kg, i.v.) was used to anesthetize the rabbits, and then, the animals were perfused transcardially with saline solution (0.9%), followed by 4% paraformaldehyde in phosphate buffer (PB, pH 7.4). After perfusion, the brains were immediately removed and cryoprotected through sequential immersion in 10%, 20%, and 30% sucrose solutions in PB. Coronal sections were then cut at 50 µm using a cryostat (Microm; Thermo Fisher Scientific Inc., Waltham, MA, USA) with serial sections collected in PB from the opening of the organum vasculosum of the lamina terminalis to the median eminence. Every fourth section was processed for PER1 labeling, following standard protocols for nuclear proteins in the rabbit brain. The sections were washed four times in PB, with each wash lasting 5 min, to clear residual aldehydes, and then incubated in a 0.5% hydrogen peroxide solution for 10 min to inhibit endogenous peroxidase activity. To reduce nonspecific antibody binding, sections were treated with 3% normal horse serum (Vector Laboratories, Inc., Burlingame, CA, USA) for 1 h at room temperature, followed by incubation at 4 °C for 48 h with a polyclonal PER1 antibody (sc-7724, Santa Cruz Biotechnology, Santa Cruz, CA, USA) at a 1:5000 dilution in 3% normal horse serum containing 0.3% Triton X-100 (Sigma, St. Louis, MO, USA).

The tissue was incubated for 1 h in biotinylated horse anti-goat serum (dilution 1:200; Vector Laboratories), followed by three PB washes, and exposed to an avidin–biotin–HRP complex (dilution 1:250; Vector Laboratories) for 1 h. Visualization of the PER1 antibody–peroxidase complex was achieved using a solution of 0.05% diaminobenzidine (DAB; Polysciences, Inc., Warrington, PA, USA) with the addition of nickel sulfate (10 mg/mL; Fisher Scientific, Pittsburgh, PA, USA), cobalt chloride (10 mg/mL; Fisher Scientific), and 0.01% hydrogen peroxide, resulting in a black-purple precipitate. The reaction was stopped after 10 min by transferring the tissue to PB.

The sections were then mounted on gelatin-coated slides, dehydrated, cleared in Hemo-De (Fisher Scientific), and coverslipped using permount to ensure consistency. All sections from the subjects at each time point were processed simultaneously. Control sections were similarly treated but without the primary antibody. The sc7724 PER1 antibody has been well characterized and validated in rabbit tissue [21,22].

### 2.4. Quantification of Immunostaining

PER1 immunoreactive cells (PER1-ir) were identified by their black-purple precipitate within the cell nuclei, produced by the DAB-nickel/cobalt reaction. Immunoreactive nuclei were quantified by assessing the labeled areas against the background optical density in an adjacent, non-immunoreactive region using IMAGE-PRO PLUS, version 5 (Media Cybernetics, Silver Spring, MD, USA). Cells exhibiting an optical density five times higher than the background level were categorized as positive for PER-1, while those below this threshold were considered negative.

PER1-ir-positive cells were counted unilaterally by two independent observers blinded to the experimental conditions, using an Olympus BX41 microscope (Olympus, Tokyo, Japan). The SCN was identified following the nomenclature of Girgis and Shi-Chang (1981) at the anteroposterior coordinate NA1 [26]. The analysis of the SCN was focused on the mid-section, with quantification carried out using a grid system as outlined in prior studies [21].

### 2.5. Statistical Analysis

Locomotion data from the GDX and Sham groups were collected from days 27 to 28 and added up into 4 h periods throughout a 24 h cycle. The values were analyzed by calculating the average of each day. These data were plotted and analyzed using two-way ANOVA to detect differences over time and between groups. For PER1-ir cell counts, two-way ANOVA was similarly applied to examine variations in cell numbers across time points within each group, with Tukey’s post hoc test conducted for further comparison (Sigma Stat, version 3.5; SPSS Inc., Chicago, IL, USA). The statistical significance was set at *p* < 0.05. Data that did not meet the homogeneity of variance assumption were rank transformed before ANOVA analysis [27]. The graph values are displayed as mean ± standard error (S.E.). Additionally, the acrophases of locomotor activity and PER1-ir cells were assessed using Cosinor analysis with ACRO.EXE software (version 3.5), following the method described by Refinetti (2006, [28]). A rhythm was considered statistically significant at *p* < 0.05.

## 3. Results

### 3.1. Effect of the Gonadectomy on Locomotor Activity

**Figure 2:**
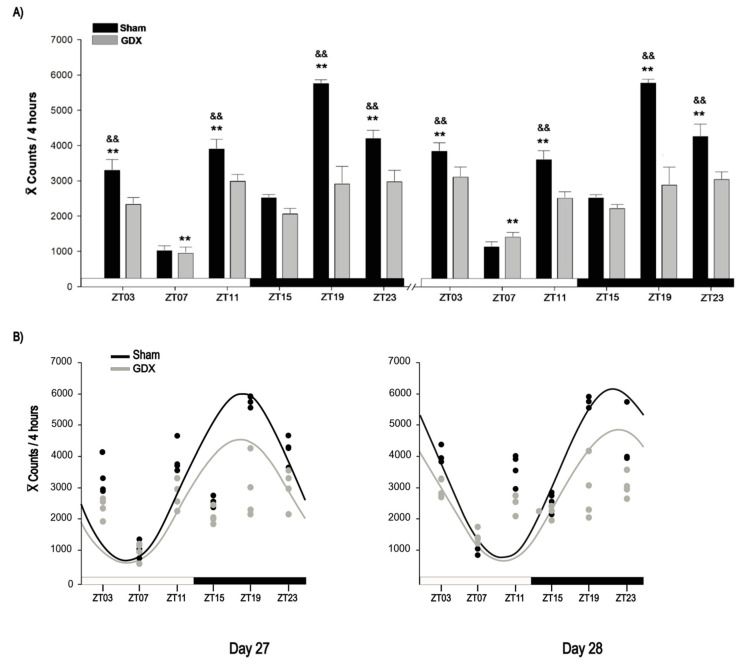
Locomotor activity of male rabbit. (**A**) Comparison of locomotor activity between Sham and GDX groups at corresponding time points at days 27 and 28. ^&&^ *p* < 0.01 indicates differences between highest locomotor behavioral activity among groups at corresponding ZT; ** *p* < 0.01 indicates differences between higher and lower values within same group. Values are presented as mean count per 4 h. Black and white bar at bottom represents light/dark (LD) condition. (**B**) Rhythmicity of locomotor activity. Cosinor analysis indicates significant rhythmicity of locomotor activity in Sham (*p* < 0.05) but not in GDX group (*p* > 0.05); grey and black circles represent individual value at respective time point; see text for details.

The Sham group exhibited a robust locomotor activity rhythm, characterized by increased activity during the dark phase, whereas the GDX group showed reduced nocturnal activity (Figure 2A). The locomotor behavior data suggested a shift in castrated rabbits; therefore, locomotor activity was analyzed at specific time points over a 24 h period for each day. On day 27, two-way ANOVA revealed a significant effect of time for both the Sham (F_5,23_ = 70.827, *p* < 0.001) and GDX (F _5,23_ = 9.079, *p* < 0.001) groups. In the Sham group, activity peaked at ZT19 and was significantly higher than at all other time points (*p* < 0.01). The activity values at ZT03, ZT11, and ZT23 were also significantly higher compared to those at ZT07 and ZT15 (*p* < 0.001, in all cases). In contrast, the GDX group showed the lowest activity at ZT07, which was significantly different from that at all other time points (*p* < 0.01), but no significant differences were observed between ZT03, ZT11, ZT15, ZT19, and ZT23 (*p* > 0.05; Figure 2A).

On day 28, two-way ANOVA revealed a similar significant effect of time in the Sham (F _5,23_ = 62.029, *p* < 0.001) and GDX (F _5,23_ = 6.420, *p* < 0.001) groups, with similar significant differences between time points on day 27. Additionally, locomotor activity in the Sham animals was higher at night, whereas the GDX group showed a decrease in locomotor activity. On both days, the Sham group activity at ZT03, ZT11, ZT19, and ZT23 was significantly higher than that in the GDX group (*p* < 0.01 in all cases). No significant differences were observed at ZT07 or ZT15 (*p* > 0.05; Figure 2A).

Cosinor analysis revealed a significant acrophase for the Sham group at 01:00 h on day 27 and at 05:00 h on day 28 (*p* < 0.001). Although the GDX group also exhibited acrophases at the same times, their rhythmicity was not statistically significant (*p* > 0.05; Figure 2B).

### 3.2. Effect of Gonadectomy on PER1 Expression in SCN

**Figure 3:**
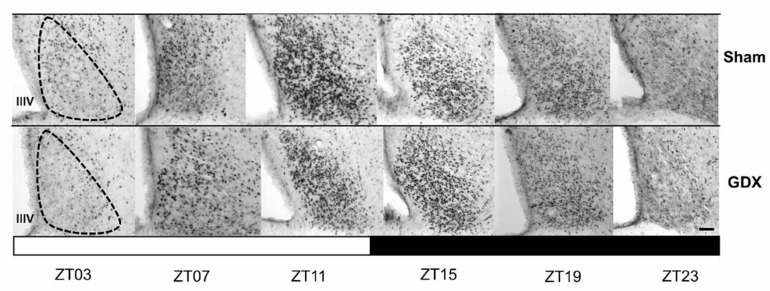
The rhythmic expression of PER1 in the suprachiasmatic nucleus (SCN) in the orchiectomized and Sham rabbits. Photomicrographs of representative sections illustrating the expression of the PER1 protein in the middle portion of the SCN at six different time points throughout a complete 24 h cycle for both groups. The black and white bar at the bottom represents the light/dark (LD) condition. The dotted line delimits the SCN; IIIV= third ventricle. Scale bar: 100 µm.

**Figure 4:**
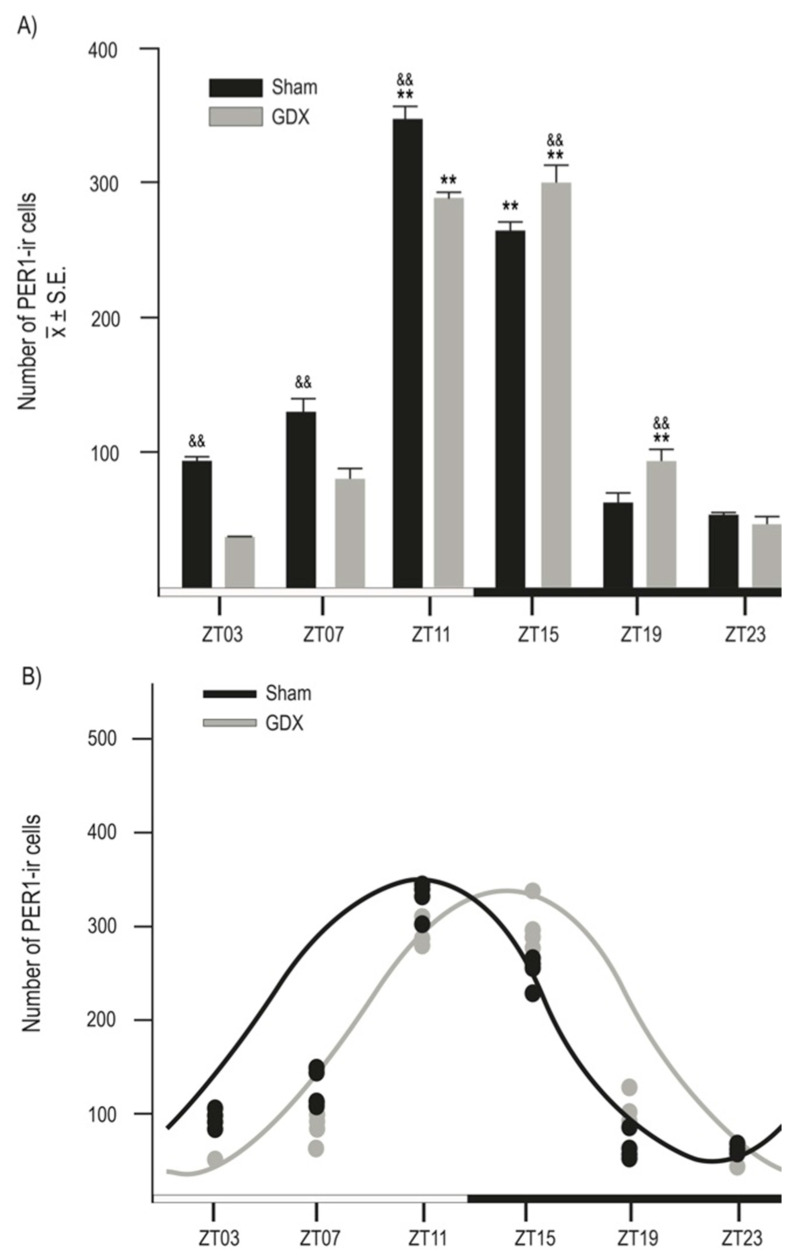
Rhythmic expression of PER1-ir cells in male rabbits. (**A**) Number of PER1-ir cells in Sham and GDX groups at six different time points throughout complete 24 h cycle. ^&&^ *p* < 0.01 indicates differences in peak values between groups at corresponding ZT. ** *p* < 0.001 indicates differences between higher and lower values within same group (see text for details). Values are presented as mean ± SEM. Black and white bar at bottom represents light/dark (LD) condition. (**B**) Cosinor analysis indicates significant rhythmicity of PER1-ir cells in both Sham and GDX groups with four-hour delay (*p* < 0.001); grey and black circles represent individual value at respective time point.

Both groups exhibited a daily rhythm of PER1-ir cells in the SCN. However, gonadectomy resulted in a reduction in the number of PER1-ir cells throughout the day. The photomicrographs in Figure 3 show PER1 protein expression in the SCN at six different time points throughout the 24 h cycle in both the Sham and GDX groups. Quantitative analysis indicated a robust rhythm in PER1-ir expression in the SCN, with the highest values observed in the evening at ZT11 and the lowest values in the late night at ZT23. ANOVA revealed a significant difference in PER1-ir cell counts over time in both the Sham (F _5,23_ = 198.045, *p* < 0.001) and GDX (F _5,23_ = 219.792, *p* < 0.001) groups. In the Sham group, the maximal values at ZT11 and ZT15 were significantly different from those at ZT03, ZT07, ZT19, and ZT23 (*p* < 0.001 in all cases). For the GDX group, the maximal values at ZT11 and ZT15 were significantly different from all other time points (*p* < 0.01 in all cases; Figure 4A). There was a significant decrease in the number of PER1-ir cells in the GDX group compared to the Sham group at ZT03, ZT07, and ZT11 (*p* < 0.01 in all cases), while an increase at ZT15 and ZT19 (*p* < 0.01 in both cases) was also observed. No significant difference was found at ZT23 (*p* > 0.05). Cosinor analysis revealed differences among acrophases for 4 h in the number of PER1-ir cells in SCN rhythmicity. The Sham group had an acrophase at 17.00 h (*p* < 0.001), whereas the GDX group had an acrophase at 21.00 h (*p* < 0.001) (Figure 4B).

## 4. Discussion

In this study, we demonstrated that gonadectomy in adult rabbits reduces locomotor activity and the expression of the clock protein PER1 in the SCN, delaying its acrophase by 4 h. The gonadectomized group, after two weeks of castration, lost their daily pattern of locomotor activity. Unlike rodents, rabbits do not have a consistent activity pattern, behaving as nocturnal animals [20,21,29]. When housed in conditions without isolation from external noise, some animals can develop diurnal habits, depending on whether the noise is in parallel with the light phase [19,30]. In the wild, rabbits demonstrate nocturnal behavior as a strategy to avoid predators [31]. However, under domestic conditions, rabbits are highly sensitive to external stimuli that synchronize their activities, such as feeding, grooming, and drinking water, among others. A study by Refinetti (2016; [30]) showed that domestic rabbits have a similar distribution of locomotor activity during the day and night, which is not as clearly defined as in rodents, which are clearly nocturnal. In the same study, rabbits exhibited interindividual variability of up to 7 h in their activity schedules, providing a wide window to shift between day and night. Thus, their schedules can adapt to management conditions [30,31].

In the present study, the animals exhibited nocturnal behavior with an acrophase between 01:00 h and 05:00 h. However, this rhythm was lost after castration, despite an unchanged lighting schedule. Differences in acrophase timing could be attributed to the interindividual variability characteristic of this species [30]. In this sense, we can propose that the decrease in androgens due to castration modifies the response to photic stimulation, inducing a delay in the acrophase of daily locomotor activity. This finding is similar to those of previous reports in rodents where castration delays the daily pattern of locomotor activity [8,10,15]. A limitation of this study is that we did not analyze the effects of castration under different light/dark schedules to determine whether these changes vary depending on the timing of the photoperiod at which the lighting stimulus is presented. In mice, it has been shown that advancing the light phase results in an increase in locomotor activity, while delaying the light phase results in a decrease in their activity [10]. Androgens influence both behavioral and physiological mechanisms in males. The testicles are the main source of these hormones, and after castration, plasma androgen levels decrease dramatically [32,33]. Androgens exert their effects through their receptors distributed in reproductive organs, such as the testicles, prostate, and seminal vesicles, as well as in tissues such as muscle, bone marrow, salivary glands, eyes, adipose tissue, spleen, and specific regions of the brain [34]. On the other hand, the effects of castration on locomotor activity are widely reported, and it has been proposed that androgens influence skeletal muscle development. Studies in rats suggest that androgens regulate muscle fiber differentiation and physiology, influencing voluntary physical activity, and castration decreases activity through direct effects on muscle without muscle mass loss, although the exact mechanisms are not yet understood [35,36,37,38]. However, central mechanisms have also been proposed where free testosterone acts on the dopaminergic system to stimulate locomotor activity [39]. Therefore, in this study, we can suggest that the decrease in locomotor activity observed in rabbits two weeks post-castration could be related to reduced plasma androgen levels. In the present study, we observed a change in the locomotor activity pattern induced by castration, resulting in the loss of daily rhythmicity. However, future studies should explore if this rhythmicity can be recovered over time. On the other hand, the effects of castration on rabbits on farms are controversial. Some studies report favorable outcomes such as better weight gain and increased market weight [40]; however, this benefit comes with drawbacks such as increased visceral weight, possibly due to the increase in abdominal adipose tissue. Conversely, other reports do not observe changes in these growth parameters but report low cortisol levels, a hormone increased during stress, which could indirectly represent improved animal welfare [41]. Metabolic and endocrine changes are widely reported in adult rabbits, including increased blood lipids and increased visceral fat accumulation [42]. Hence, under companion animal conditions, careful attention to diet and environmental enrichment is necessary to promote exercise. Castration remains a viable practice for housing rabbits in groups, as it encourages social interaction, reduces aggressive behavior, and facilitates the provision of shelters [43].

The SCN is the master clock of the circadian system and primarily synchronizes circadian rhythms through photic signals from the environment [44]. However, non-photic signals also influence synchronization mechanisms [45,46]. Endocrine signals such as androgen secretion can modulate this rhythmicity [10,15,47]. The PER1 protein has a crucial role in the molecular clock mechanism, as it forms a dimer with CRY, which is part of a feedback loop that regulates its own transcription by inhibiting the expression of CLOCK and BMAL. This, in turn, controls the expression of clock-controlled genes [48,49].

It is of interest to observe how castration decreased PER1 expression in the SCN and induced a delay in its acrophase. In previous studies, we have shown that the SCN of neonatal and adult rabbits exhibits a daily rhythm of PER1 expression, with peak levels at the end of the light phase [21,22]. Exposure to light at specific times of the day induces PER1 expression in the SCN of rodents [50,51]. However, it has been shown that non-photic stimulation throughout the day, such as induced activity on a novel running wheel [52] or hypocaloric feeding [53], decreases clock gene expression. A decrease in androgens is a factor that influences clock gene expression in mice in response to light stimuli. However, the mechanisms are still unclear, as it has been shown in mice that Per2 expression decreases when the photic stimulus is applied during the early light phase, but not when applied during the late light phase [15].

## 5. Conclusions

In conclusion, castration in rabbits modifies their daily rhythm of locomotor activity, which could be modulated by synergism between the effects of the systemic absence of androgens at the central level on the master clock (SCN) and at the peripheral level through its action on muscle tissue.

## Data Availability

The raw data supporting the conclusions of this article will be made available by the authors on request.
